# Supplementary material for: Mechanisms governing the pioneering and redistribution capabilities of the non-classical pioneer PU.1
Source: Nat Commun. 2020 Jan 21;11:402. doi: 10.1038/s41467-019-13960-2 (PMC6972792; doi:10.1038/s41467-019-13960-2)
Supplement: Supplementary file 7 — Source data [file 41467_2019_13960_MOESM7_ESM.zip › Source_Data/FigureS2/FigureS2B_MotifScanOutput/KmeansCluster13/homerResults/motif12.similar.html]

motif12

## Information for motif12

A
C
G
T
T
A
C
G
C
G
T
A
A
T
C
G
A
C
G
T
A
T
G
C
C
G
T
A
A
C
T
G
G
T
A
C
G
A
T
C
T
G
C
A
  
Reverse Opposite:  

A
C
G
T
C
T
A
G
C
A
T
G
T
G
A
C
A
C
G
T
A
T
C
G
G
T
C
A
A
T
G
C
A
C
G
T
A
T
G
C
C
G
T
A
  

|  |  |
| --- | --- |
| p-value: | 1e-32 |
| log p-value: | -7.427e+01 |
| Information Content per bp: | 1.675 |
| Number of Target Sequences with motif | 141.0 |
| Percentage of Target Sequences with motif | 8.33% |
| Number of Background Sequences with motif | 1111.4 |
| Percentage of Background Sequences with motif | 2.46% |
| Average Position of motif in Targets | 97.1 +/- 52.0bp |
| Average Position of motif in Background | 98.7 +/- 62.4bp |
| Strand Bias (log2 ratio + to - strand density) | 0.1 |
| Multiplicity (# of sites on avg that occur together) | 1.03 |
| Motif File: | file (matrix) reverse opposite |

### Similar de novo motifs found

|  |  |  |  |  |  |  |  |
| --- | --- | --- | --- | --- | --- | --- | --- |
| Rank | Match Score | Redundant Motif | P-value | log P-value | % of Targets | % of Background | Motif file |
| 1 | 0.965 | C G A T T G A C A C G T A C T G C G T A A T C G A C G T A G T C C G T A A C T G | 1e-27 | -64.222925 | 6.86% | 1.95% | motif file (matrix) |
| 2 | 0.937 | A G C T A C T G C G T A A T C G A C G T A G T C C G T A A T G C | 1e-27 | -63.522059 | 7.62% | 2.37% | motif file (matrix) |
| 3 | 0.951 | T A C G T A G C A G C T A T C G G T C A T A C G C G A T T G A C C T G A | 1e-26 | -62.133084 | 9.22% | 3.33% | motif file (matrix) |
| 4 | 0.817 | C G A T A C T G G C T A A T G C G C A T A G T C C G T A T G A C G T A C T G A C C G T A T A C G T G C A | 1e-19 | -45.718979 | 3.01% | 0.54% | motif file (matrix) |
| 5 | 0.788 | C T A G A T G C G C A T T A C G G C T A A T G C G C A T A T G C G T C A T A G C G T C A T A C G | 1e-18 | -41.986802 | 5.08% | 1.60% | motif file (matrix) |
| 6 | 0.781 | A C G T A T C G C G T A A T G C C G A T A G T C G T C A T G A C G A T C T G A C G T C A A T C G | 1e-16 | -37.365012 | 2.72% | 0.55% | motif file (matrix) |
